# Scalp trauma in lichen planopilaris: Case reports of disease progression from fire fighter helmet use

**DOI:** 10.1016/j.jdcr.2024.04.016

**Published:** 2024-04-21

**Authors:** Ambika Nohria, Deesha Desai, Camila Ortiz, Shadi Khalil, Ata Moshiri, Jerry Shapiro, Kristen Lo Sicco

**Affiliations:** a The Ronald O. Perelman Department of Dermatology, NYU Grossman School of Medicine, New York, New York; b University of Pittsburgh School of Medicine, Pittsburgh, Pennsylvania

**Keywords:** fire fighter, helmet, lichen planopilaris, occupational hazard, scalp trauma

## Introduction

Lichen planopilaris (LPP) is an inflammatory hair loss condition resulting in scarring alopecia. Classic LPP presents with scarred alopecic patches most commonly on the vertex and parietal scalp.^1^ Patients, most frequently women aged 25 to 70, may also experience symptoms including pruritus, pain, or scaling.^1^

The pathomechanism behind LPP remains incompletely understood; however, it is proposed to result from a T-lymphocyte driven autoimmune attack of the bulge region of the hair follicle which harbors multipotent cells responsible for hair regeneration.^2^ Damage to this region leads to follicular scarring and prevention of future hair growth.^2^ In addition to genetic predisposition, environmental triggers have been proposed to contribute to LPP including drugs, viruses, and contact sensitizers.^1^ Importantly, physical scalp trauma has also been associated with LPP.^3^

Herein, we describe 2 cases of firefighters who report frequent use of fire helmets worsening their LPP. Additionally, both patients have a remote history of hair transplantation, which may also have contributed to the development of LPP. We propose that the prolonged use of a fire helmet represents a novel example of scalp trauma resulting in progression of LPP.

## Case reports

### Case 1

**Fig 1:**
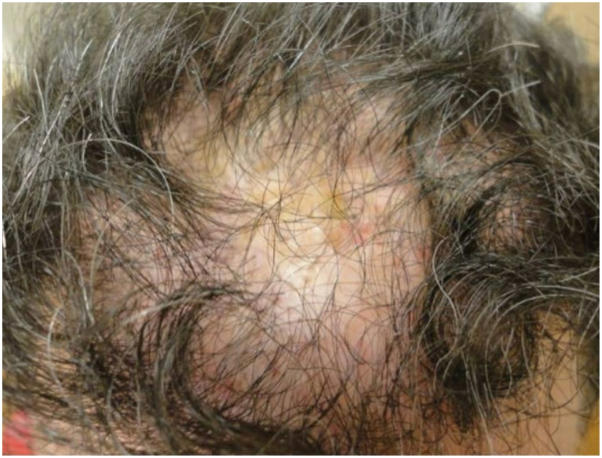
Initial presentation in 2013 of lichen planopilaris on vertex of scalp (case 1).

**Fig 2:**
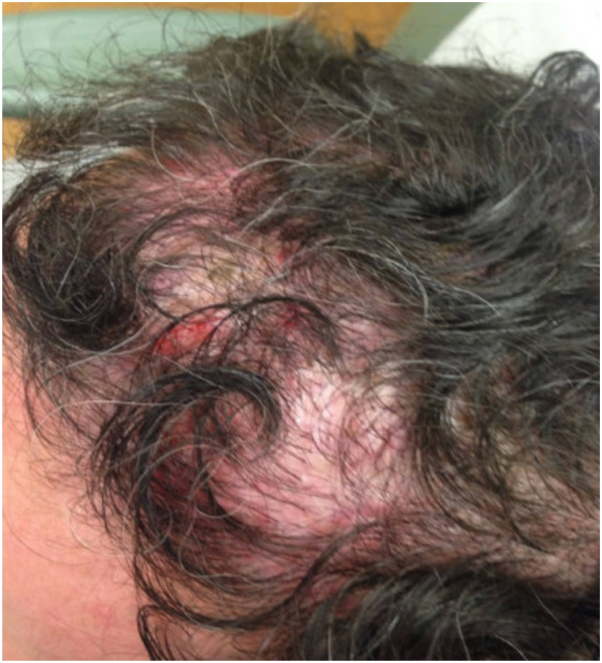
Flare of lichen planopilaris associated scalp symptoms in 2015 despite over 1 year of medical therapy (case 1).

A 47-year-old male presented to the office in 2013 endorsing 20 years of hair loss (Fig 1). The patient works actively as a New York City fire fighter. He reports previous evaluation for androgenetic alopecia (AGA) and a history of 4 hair transplants, most recently in 2003. Due to progressive hair loss, the patient was evaluated by his hair transplant surgeon who raised concern for scarring alopecia and performed a scalp biopsy which demonstrated LPP. After consultation at New York University Langone Health, he was started on a treatment protocol consisting of topical clobetasol 0.05% twice a day, hydroxychloroquine 400 mg/day, finasteride 1 mg/day, and intralesional triamcinolone acetonide injections once/month. The patient was also advised to avoid excessive scalp friction. Despite adherence to medical therapy, over the course of 10 years of treatment he experienced intermittent disease flares including pruritus, erythema, scaling, and crusting (Fig 2). Notably, he reports feeling “uncomfortable” when wearing his firefighting helmet. He also developed 2 squamous cell carcinomas on the vertex and occipital scalp in October of 2019 and 2020 at least likely in part due to chronic inflammation from LPP. Both were treated with Mohs excision.

### Case 2

**Fig 3:**
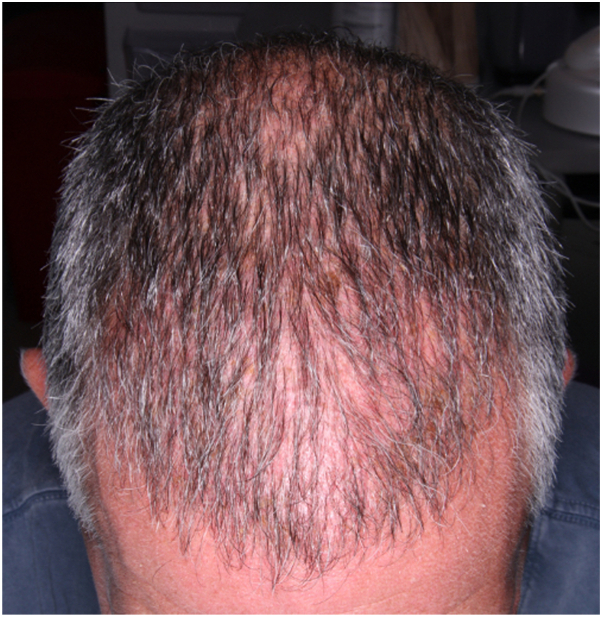
Frontal hairline erythema and crusting present in 2023 (case 2).

**Fig 4:**
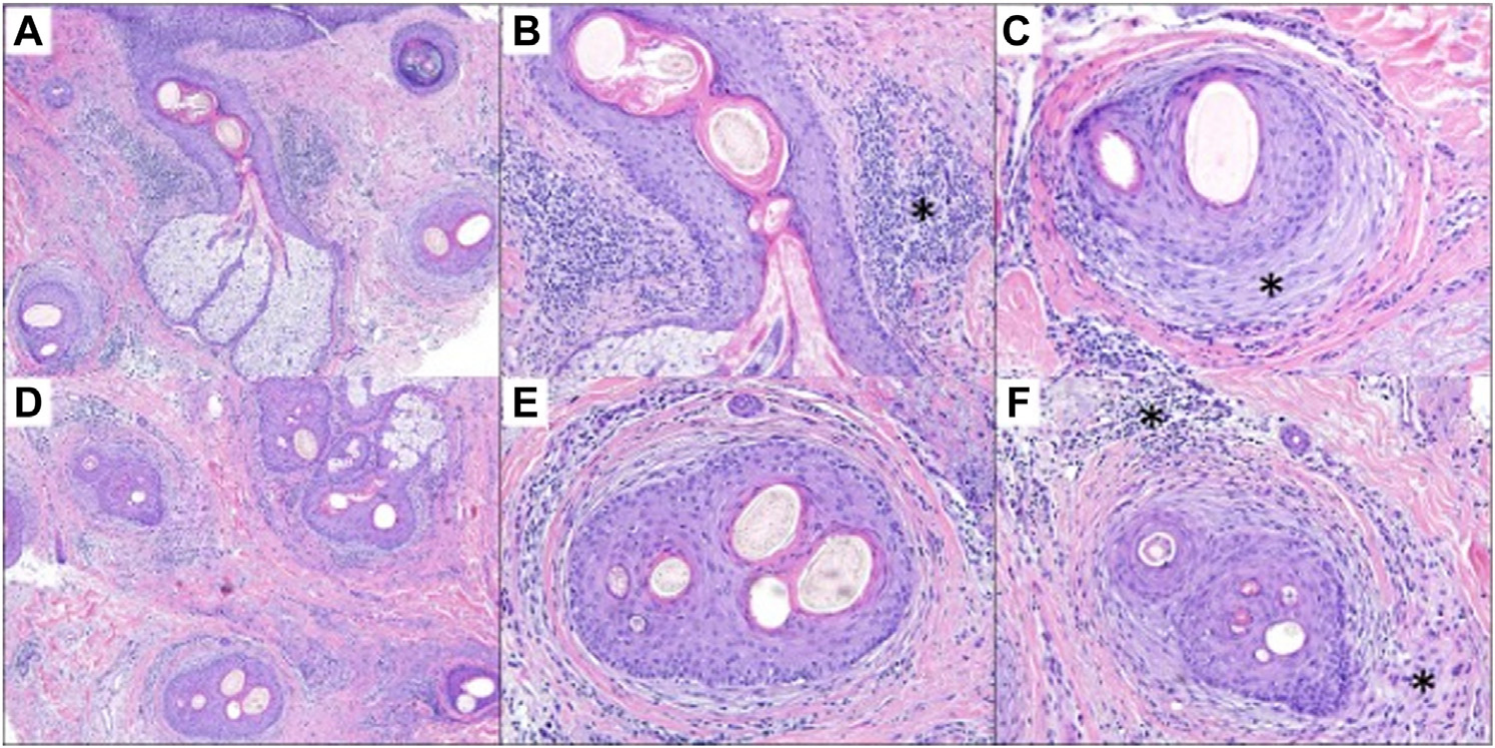
Histopathologic evaluation of a scalp biopsy specimen (case 2). **A,** Lichenoid interface dermatitis of a central follicle with prominent perifollicular fibroplasia and scar. **B,** Higher-power magnification of a lichenoid dermatitis (*asterisk*) surrounding the isthmus of the hair follicle. **C,** Prominent concentric fibroplasia (*asterisk*) of the hair follicle with active lymphocytic inflammation. **D,** Reduced numbers of follicular units with inflammation and scarring of the dermis. **E,** Close-up of a follicular unit demonstrating polytrichia, fibroplasia, with terminal hairs of various calibers and active lymphocytic inflammation. **F,** Polytrichia with miniaturized hairs in association with an inflammatory infiltrate composed of lymphocytes and histiocytes (*asterisk*).

A 44-year-old male presented to the office in 2023 reporting 10 years of hair loss (Fig 3). The patient works actively as a New York City fire fighter. He reports a prior history of 3 hair transplants, most recently in 2018. In May of 2022, the patient underwent a skin punch biopsy of the central frontal scalp which demonstrated miniaturization of terminal hairs consistent with AGA. However, he continued to have persistent scaling, itching, and discomfort of the scalp, which prompted a repeat skin punch biopsy 4 months later of the right central parietal scalp. Histopathologic analysis of the sample demonstrated a follicular interface dermatitis with perifollicular fibroplasia, consistent with a diagnosis of LPP (Fig 4). The patient was started on new treatments including a compounded topical solution containing tacrolimus 0.3%, clobetasol 0.05%, and minoxidil 5% twice a day, naltrexone 3 mg/day, pioglitazone 1 mg/day, hydroxychloroquine 400 mg/day, and Excimer 308 nm laser therapy twice/week. He was also instructed to continue his prior use of ketoconazole 2% shampoo 3 times/week, finasteride 1 mg/day, minoxidil 5 mg/day, and intralesional triamcinolone acetonide injections once/month. Six months later, he presented for follow-up and, despite compliance with medical therapy, reported further hair loss and persistent scalp symptoms. The patient made note at this visit that his symptoms were significantly worse during working hours and that the use of his firefighting helmet aggravated his scalp.

## Discussion

These 2 cases highlight a unique occupational exposure—a fire helmet—contributing to LPP progression through physical scalp trauma. Fire helmets typically weigh 3 to 4 pounds and can exert significant pressure on the scalp with prolonged use. Frequent donning and doffing of the helmet also represents a source of frictional trauma. Both patients described here report significant worsening of their symptoms associated with helmet use, suggesting this exposure contributed to LPP progression.

Prior case reports have demonstrated the potential for other forms of scalp trauma to contribute to LPP including long term use of a high-tension hairstyle, direct injury to the scalp, and a patient who frequently spun on his head during breakdancing.^3^, ^4^, ^5^ Reports have also been published on LPP developing after wig use, which is important to consider as patients with LPP may utilize wigs as cranial prostheses for camouflage.^6^ Although incompletely understood, it is posited that frictional trauma may contribute to LPP by inducing an inflammatory environment that disrupts the normal immune protection of the hair follicle.^7^ Others suggest that trauma downregulates peroxisome proliferator–activated receptor γ signaling which has been linked to LPP.^8^

Interestingly, both cases report a remote history of multiple hair transplants prior to LPP diagnosis. It is well documented that hair transplantation can elicit or worsen LPP, and patients are generally advised to only consider hair transplant if disease quiescence has been sustained off medical therapy.^7^^,^^9^ Further research is warranted to determine whether multiple hair transplants contribute more significantly to the development of LPP compared to just one procedure. While hair transplantation may have contributed toward the development of LPP, the progression and persistence of symptoms is more likely attributed to fire helmet use due to the temporal association.

Notably, the second patient described here initially was biopsy diagnosed with AGA. Many patients with scarring alopecia may concomitantly experience AGA, requiring treatment tailored at managing both conditions. LPP may also mimic AGA in a presentation termed fibrosing alopecia in a pattern distribution. Biopsy may prove essential in these cases for distinguishing between the two.^10^

Identification of the fire helmet as a source of scalp trauma is a new finding in LPP. Providers must take a thorough social history when evaluating patients with hair loss to identify possible environmental, including occupational, factors contributing to disease. Patients with LPP should be counseled to avoid trauma to the scalp wherever possible.

## Conflicts of interest

Dr Shapiro is a consultant for Lilly, Replicel Life Sciences, Thirty Madison, and DS Laboratories. Drs Shapiro and Lo Sicco have been investigators for Regen Lab and are investigators for Pfizer. Dr Lo Sicco is a consultant for Pfizer and Aquis. Authors Nohria, Desai, Drs Ortiz, Khalil, and Moshiri have no conflicts of interest to declare.
